# Evaluation of efficacy of combination of methotrexate and hydroxychloroquine with leflunomide in active rheumatoid arthritis

**DOI:** 10.4103/0253-7613.71916

**Published:** 2010-12

**Authors:** N.S. Shashikumar, M.C. Shivamurthy, S. Chandrashekara

**Affiliations:** Department of Pharmacology, Mandya Institute of Medical Sciences, Mandya, India; 1 Department of Pharmacology, M S Ramaiah Medical College, Bangalore, India; 2 Department of Rheumatology and Immunology, M S Ramaiah Medical College, Bangalore, India

**Keywords:** DAS_28_, hydroxychloroquine, leflunomide, methotrexate, rheumatoid arthritis

## Abstract

**Background:**

Several new drugs for rheumatoid arthritis are available including leflunomide. Comparative studies of treatment with leflunomide (against methotrexate) report a better quality of life.

**Aim:**

This study was designed to evaluate the efficacy of combination of methotrexate and hydroxychloroquine with leflunomide, a new disease modifying antirheumatoid drug. Analysis was of intent to treat group.

**Materials and Methods:**

This was an open labeled, randomized, comparative clinical trial in the department of rheumatology and immunology, at a tertiary care center in Bangalore. Patients who have diagnosed with rheumatoid arthritis as per American College of Rheumatology aged between 18 and 60 years were recruited and randomized to receive leflunomide (10 mg/day p.o.) or a combination of methotrexate and hydroxychloroquine (7.5 mg/week p.o. and 200 mg/day p.o., respectively) along with folate supplementation for 12 weeks. The European League Against Rheumatism criteria of improvement according to disease activity score 28 was considered as the primary efficacy variable. Baseline and end of study values were evaluated. The duration of the study period was 1 year. Analysis of variance (ANOVA) and Wilcoxon Signed rank test were used for statistical analysis.

**Results:**

After 12 weeks, improvement noted in patients treated with leflunomide was similar to those treated with a combination of methotrexate and hydroxychloroquine. There was no statistical significance in improvement in disease activity between the two groups (*P* = 0.377).

**Conclusion:**

Combination of methotrexate and hydroxychloroquine is equivalent to leflunomide in terms of efficacy in reducing disease activity in the initial treatment of severe rheumatoid arthritis.

## Introduction

Complications of rheumatoid arthritis may begin to develop within months of presentation. Eventually, multiorgan systems may be affected.[1] If untreated, it ultimately causes functional impairment and 20–30% of persons with rheumatoid arthritis become permanently work-disabled within 2 to 3 years of diagnosis.[2]

Studies have shown that treatment with leflunomide frequently causes adverse drug reactions. More than half of the patients withdraw from leflunomide treatment within 1 year because of its adverse effects.[3] Another study has concluded that hydroxychloroquine increased the potency of methotrexate and also sustained its effect.[4] Data from the UK have shown that if conventional disease-modifying antirheumatoid drugs (DMARDs) are optimized and used in combination, control of the disease can be achieved in more than half of patients who otherwise will be candidates for newer promising agents.[5]

Many new drugs are introduced in India for the treatment of rheumatoid arthritis; however, their efficacy is yet to be established. Hence, studies are necessary in Indian patients to compare the efficacy with methotrexate, an age old, gold standard DMARD. This study evaluates the efficacy of the combination of methotrexate and hydroxychloroquine against leflunomide, a new DMARD as initial therapy in severe active rheumatoid arthritis. Hydroxychloroquine is known to increase the potency of methotrexate as well as increase its safety profile and reduce the dose.[4]

## Materials and Methods

This was a randomized, open labeled, comparative clinical trial conducted between January and December 2006 at the department of rheumatology and immunology of a tertiary care center in accordance with the declaration of Helsinki following approval of Institutional Ethics Committee. Informed consent was obtained from all the patients. A brief history was taken, and general examination was performed. Patients of either sex between the age group of 18 and 60 years with active rheumatoid arthritis according to American College of Rheumatology (ACR) criteria that conformed to European League Against Rheumatism (EULAR) DAS score of 5.1 and above were recruited. Patients with moderate-to-severe hepatic, renal, pulmonary, endocrine and hematological disorder, uncontrolled hypertension, pregnant or lactating patients and those belonging to reproductive age, and not willing to practice contraception were not included in the study.

Sixty patients were randomly allotted into two groups of 30 patients in each. Random numbers were generated from online software www.researchrandomiser.com Baseline blood investigations were performed, and the EULAR criteria of improvement was considered as the primary efficacy variable.[2,6] Patients were assessed for severity of disease with DAS_28_ after 6 and 12 weeks of initiating therapy with study drugs. The hematological and clinical adverse events were the secondary parameters. DAS_28_ was calculated using the following formula: DAS_28_ = 0.56 × sqrt (tender 28) + 0.28 × sqrt (swollen 28) + 0.70 × ln (ESR) + 0.014 × GH. The score of DAS_28_ indicates the current activity of the rheumatoid arthritis. Patients with DAS_28_ score of <3.2, 3.2-5.1, and >5.1 were considered to have low, moderate, and high disease activity, respectively. DAS_28_ score <2.6 indicates remission. Finally, the improvement in disease activity is measured by calculating the difference in DAS_28_ scores of baseline with post 6 and 12 weeks scores.

Patients in group-1 received a combination of drugs, namely tablet methotrexate 7.5 mg/week p.o. and tablet hydroxychloroquine 200 mg p.o. daily. Group-2 received tablet leflunomide 10 mg p.o. daily. Loading dose of leflunomide was not utilized as efficacy ranging from 50% to 87% with monotherapy has been observed in various situations irrespective of the absence of loading dose.[7–10] Dose escalation was not done as the addition of hydroxychloroquine reduces the dose of methotrexate and effects start after 6–12 weeks. Hematological parameters assessed included hemoglobin, packed cell volume, red blood cell count, white blood cell count, platelet count, creatinine, aspartate aminotransferases, and alanine aminotransferases. Blood pressure was recorded at the baseline, 6 and 12 weeks.

### Statistical methods

The data were analyzed with analysis of variance (ANOVA) and Wilcoxon signed-rank test. Values are expressed as numbers, percentages, and mean ± SD. ANOVA was used to analyze the variables within the groups and Wilcoxon signed-rank test to find the significance between groups. Significant figures for Wilcoxon signed-rank test were: P < 0.10 —suggestive significance, *P* ≤ 0.05—moderately significant, and *P* ≤ 0.01—strongly significant. The statistical software namely SPSS 11.0 and Systat 8.0 were used for the analysis of the data.

## Results

Out of 126 patients screened, 60 new patients with active rheumatoid arthritis according to ACR criteria that conformed to EULAR, DAS score of 5.1, and above were recruited. Intention to treat analysis included 60 patients of which 90% were females. The distribution of subjects was even across all the five decades of age falling between the age limits for recruitment into the study. An increased incidence of disease was seen in the female study subjects (90% vs. 10% of male subjects). The baseline demographic parameters did not vary between the two groups [Table 1].

**Table 1:** Baseline demographic data and clinical characteristics of the patients

*Characteristics*	*MTX + HCQ (Group 1)*	*LEF (Group 2)*	*P value*
Number of patients recruited	30	30	
Number of patients at follow-up	30	29	
Female sex (%)	90	86.66	
Age (years)	48.24 ± 11.44	49.33 ± 11.381	0.683
Total joint count	19.03 ± 10.61	19.86 ± 9.22	0.669
Swollen joint count	8.56 ± 9.94	10.62 ± 9.90	0.306
Erythrocyte	40.70 ± 30.62	42.82 ± 23.90	0.569
sedimentation rate			
Pain assessment on	82.50 ± 12.52	88.88 ± 6.08	0.064
Visual Analog Scale			
Disease Activity Score	6.42 ± 1.74	6.91 ± 1.19	0.453

Data shown is mean ± SD;

*Statistically significant; Mtx = Methotrexate; HCQ = hydroxychloroquine; LEF = leflunomide.

EULAR criteria of disease activity showed improvement in a significant number of patients treated with methotrexate and hydroxychloroquine combination as well as in the patients treated with leflunomide. Disease activity from high status was reduced to moderate status after 12 weeks therapy in both the groups. Disease activity score within the groups decreased, while it did not show any statistical significance between the groups [Tables 2 and 3]. Similarly, the improvement in disease activity within the groups decreased while it did not show any statistical significance between the groups [Table 4]. The hematological and clinical adverse events were the secondary parameters. One subject presented with severe vomiting on day 3 after initiation therapy with leflunomide and was withdrawn from the study as plasma liver enzymes were elevated (SGOT, 1500 IU and SGPT, 1340 IU). Assessment of liver enzymes in other patients in both the groups after 6 and 12 weeks did not show much variation. Hematological parameters assessed after 6 and 12 weeks were also within normal limits in both the groups. The most common adverse effects were nausea and alopecia. Comparison between the two groups did not show statistical significance. There was no effect on comorbid conditions.

**Table 2:** Comparison of various parameters of disease activity score

	*MTX + HCQ (Group 1)*				*LEF (Group 2)*			
	*Baseline*	*Sixth week*	*Twelfth week*	*P*	*Baseline*	*Sixth week*	*Twelfth week*	*P*
TJC	19.03 ± 10.61	8.46 ± 8.70	15.03 ± 9.08	0.000*	19.86 ± 9.22	7.96 ± 6.36	14.89 ± 7.80	0.000*
SJC	8.56 ± 9.94	7.37 ± 9.06	7.90 ± 9.33	0.000*	10.62 ± 9.90	8.41 ± 8.49	9.58 ± 9.32	0.000*
ESR	40.70 ± 30.62	3.00 ± 38.49	28.50 ± 29.23	0.000*	42.82 ± 23.90	14.37 ± 18.26	2924 ± 22.01	0.000*
PVAS	82.50 ± 12.52	17.87 ± 16.87	54.10 ± 12.61	0.000*	88.88 ± 6.08	23.45 ± 14.29	58.58 ± 8.19	0.000*

Data shown is mean ± SD;

* Statistically significant; Mtx = Methotrexate; HCQ = hydroxychloroquine; LEF = leflunomide; TJC = total joint count; SJC = swollen joint count; ESR = erythrocyte sedimentation rate; PVAS = pain as on Visual Analog Scale.

**Table 3:** Comparison of disease activity score (mean ±SD)

	*MTX + HCQ (Min.-Max.)*	*LEF (Min.-Max.)*	*P*
Baseline	6.42 ± 1.74 (VA) (1–8)	6.91 ± 1.19 (VA) (3–7)	0.453
Sixth week	5.00 ± 1.61 (MA) (1–8)	5.30 ± 1.28 (MA) (3–7)	0.601
Twelfth week	3.37 ± 0.84 (MA) (1–5)	3.61 ± 0.63 (MA) (2–5)	0.266
*P*	<0.001*	<0.001*	

Disease activity: VA, very active; MA, moderate active;

* Statistically significant; MTX = Methotrexate; HCQ = hydroxychloroquine; LEF = leflunomide

**Table 4:** Improvement in disease activity score (mean ± SD) over 12 weeks

*Duration*	*MTX + HCQ*	*LEF*	*P*
6 weeks	1.42+1.61 (MI)	1.61+1.28 (MI)	0.601
12 weeks	3.04+1.29 (MI)	3.29+0.79 (MI)	0.377
*P*	<0.001*	< 0.001*	

* Statistically significant; MI = Moderate improvement; MTX = methotrexate; HCQ = hydroxychloroquine; LEF = leflunomide.

## Discussion

This study was designed to evaluate the efficacy of the combination of methotrexate and hydroxychloroquine with leflunomide in the treatment of active rheumatoid arthritis over a period of 12 weeks.

A significant change in disease activity score was seen in both the groups at 6 and 12 weeks after initiating therapy with the investigative drugs. The study has highlighted the improvement in disease activity score in group-1 and group-2 by 3.04 and 3.29, respectively. However, the comparison of disease activity score in between groups was insignificant (*P* = 0.377). Hence, it may be concluded that the group treated with leflunomide did not have an advantage over those treated with combination of methotrexate and hydroxychloroquine. The study showed that the combination of methotrexate and hydroxychloroquine is equivalent to leflunomide in improving the disease activity and is similar in the hepatic safety profile. We conclude that methotrexate in combination with hydroxychloroquine is equal in efficacy to new drug leflunomide as an initial therapy in severe active rheumatoid arthritis.

Hematological parameters showed no change. In addition, there was no variation in liver enzymes in both the groups leading to a conclusion that during this 12-week study, the tested drugs did not show a hepatic toxicity. The purpose of adding hydroxychloroquine is to reduce the dose of methotrexate, thereby reducing its hepato-toxic effects and this too was substantiated. Earlier studies have shown liver enzyme elevation, which subsequently normalized.[11,12]

The outcome of the study has a pronounced effect regarding the direct cost of therapy involved in treating rheumatoid arthritis. Treatment for severe active rheumatoid arthritis with combination of methotrexate and hydroxychloroquine is economical and can be considered an effective and safe option in the Indian population against leflunomide, an expensive drug. This study needs to be validated with larger study involving greater number of subjects for longer duration and follow-ups.
